# Differential DNA methylation profiling of spermatogenesis-associated genes in patients with idiopathic hypospermatogenesis: a hospital-based case-control study

**DOI:** 10.3389/frph.2026.1932019

**Published:** 2026-10-28

**Authors:** Nisha Sharma, Sonali Singh, Vivek Kumar, Ashutosh Halder, Seema Kaushal, Manoj Kumar, Manish Jain

**Affiliations:** 1 Department of Reproductive Biology, All India Institute of Medical Sciences, New Delhi, India; 2 Department of Pathology, All India Institute of Medical Sciences, New Delhi, India; 3 Department of Urology, All India Institute of Medical Sciences, New Delhi, India

**Keywords:** differential methylation profiling, DNA methylation, idiopathic hypospermatogenesis, non-obstructive azoospermia, spermatogenesis genes

## Abstract

Hypospermatogenesis is a histopathological subtype of non-obstructive azoospermia and is characterized by a decrease in the total number of germ cells within the seminiferous tubule as a result of spermatogenic failure. Germ cell epigenetic reprograming during development shows that the methylation status of testicular DNA is highly distinct in which hypomethylated foci predominates. A unique testicular DNA methylation pattern needs to be established for various clinical and histopathological subtypes of azoospermia, in order to delineate the epigenetic factors associated with the condition. This study aim to explore the differential methylation profile in idiopathic hypospermatogenesis patients using methylation microarray technique from testicular tissue DNA. A comprehensive clinical history, blood sample, semen analysis parameters, and reproductive endocrine evaluation reports of 51 hypospermatogenesis patients were collected. The known genetic causes were investigated for exclusion using XY fluorescent *in-situ* hybridization and Yq microdeletion techniques. DNA methylation microarray was performed, on 40 idiopathic hypospermatogenesis and 10 normospermatogenesis patient to identify differential DNA methylation patterns in spermatogenesis associated genes. A genome-wide differential DNA methylation profiling of 40 hypospermatogenesis patients identified 1,666 differentially methylated regions localised predominantly at promotor regions. A significant proportion (70%) of these differentially methylated genomic regions exhibited hypermethylation, suggesting widespread transcriptional repression of spermatogenesis-associated genes. The transcription factor protein-protein interaction analysis identified six clusters viz., TBP, AR, SP3, TP53, CCNT2 and SIN3A, indicating their potential role in the epigenetic mechanisms of the clinical condition of male infertility. Differential methylation burden analysis showed 4/29 high burden and 5/29 intermediated burden patients. Functional enrichment analysis of promoter-associated differentially methylated spermatogenic genes were found implicated in crucial molecular functions including RNA binding, DNA binding, transcription regulation, and spermatid differentiation. The findings suggest that male infertility may, in part, be driven by the silencing of genes that are not just transcriptionally active, but that also act as nodal regulators in transcription factor complexes essential for germ cell viability, hormone responsiveness, and sperm maturation. Our analysis also demonstrated methylation aberrations display a non-uniform distribution, highlighting patient-specific epigenetic patterns, suggesting a high degree of inter-individual variability in the epigenomic contributors to hypospermatogenesis.

## 1 Introduction

Hypospermatogenesis (HS) is one of the frequently observed histopathological subtype of non-obstructive azoospermia (NOA), comprising approximately 43% of cases, it is also associated with highest sperm retrieval rates (1, 2). HS is regarded as a mild phenotype of primary testicular failure and is hypothesised to be originating from defects within the spermatogonial stem cell population (3). As per the human protein atlas data, approximately 932 genes can be classified as testis-enriched, highlighting the highly specialized transcriptional landscape required for normal spermatogenesis (4, 5). Aberrant DNA methylation and demethylation are increasingly recognized as key epigenetic mechanisms underlying impaired spermatogenesis in NOA. Most studies have primarily focused on global methylation changes and dysregulation of core methylation machinery. (6, 7). Alongside DNA methyltransferases, active demethylation mediated by the TET family genes has been previously explored in NOA, with studies reporting altered 5mC and 5hmC patterns in testicular tissues (8–10). Studies have reported aberrant DNA methylation in several genes associated with spermatogenesis and male infertility, including *MTHFR, DDR1, SPATA16, CNGA1, MAEL, BOLL, DDX4, and SOX30 (*11, 12). Previous genome-wide DNA methylation studies in NOA have also highlighted the importance of imprinting-associated genes along with alterations in CCCTC- binding factor (CTCF) -binding regions, suggesting that disrupted imprinting and epigenetic regulation may contribute to spermatogenic failure (13, 14). DNA methylation has been previously studied in NOA patients both at the level of blood sample and testicular tissue sample derived DNA. Ferfouri et al. reported aberrant methylation across 38 genes implicated in spermatogenesis, including *PDHA2, PARP12, FHIT, RPTOR, GSTM1, and HDAC4*, alongside several testis-enriched genes such as *NFATC1, NMNAT3, and PIAS2 (*15). Similarly, Wu et al. demonstrated hypermethylation of *DAZL* and differential methylation in key germ-cell regulatory genes including *NR5A1, TOPAZ1, and DND1* (16). In addition, Li et al. identified hypermethylated loci within the *CDK* gene family along with hypomethylation of genes such as *CECR2, GRAP2, CCL23, SLA2, and ZCCHC13* (17). Together, these findings underscore the significant contribution of DNA methylation abnormalities in disrupting spermatogenic pathways and reinforce the importance of investigating testis-enriched gene methylation landscapes in NOA. Despite the growing body of evidence supporting the role of epigenetic defects in male infertility, the prevalence and specificity of such abnormalities in patients with idiopathic HS remain under-characterised. Therefore; the current study has been performed to identify the aetiological factors in idiopathic cases of HS with an exploratory approach.

## 2 Materials and methods

### 2.1 Patient recruitment and laboratory procedures

The study was conducted at the Department of Reproductive Biology, All India Institute of Medical Sciences, New Delhi, India, for three years from March 2020 to March 2023. Infertile male patients coming to the Department of Urology were recruited, and clinical history and physical examination were performed for each case. Informed written consent was obtained from the participants, and the study has been performed according to the Declaration of Helsinki. The research was conducted according to the guidelines of the institute ethical committee of the All India Institute of Medical Sciences in Delhi, India. The approval ID is IEC-151/06.03.2020, RP-17/2020. Hormonal evaluation of infertile men was performed using chemiluminescent microparticle immunoassay (CMIA). Quantification of serum follicle stimulating hormone (FSH), serum luteinizing hormone (LH), and serum testosterone was performed using 2nd generation Abbott laboratories’ kits. Semen analysis was performed for each infertile man following WHO criteria 2021 (18). The ejaculate was collected in a preweighed container, and semen volume was calculated. A 30-minute incubation for liquefication of the semen sample was done, and the pH was determined. The semen sample was heated in strong acid and resorcinol to measure fructose concentration. A 20 µL semen sample was prepared by carefully mixing the ejaculate and examined under 10 ×  followed by 40 ×  magnifications. For spermatozoa >= 101 per field at 40 ×  magnification, a 1:20 dilution ratio was chosen to count sperm in 50 µL of well mixed ejaculate and 950 µL of fixative. Microscopic counting was done in Neubauer's chamber. Cases with no sperm seen even after centrifugation and a sperm count < 2.5 mio/mL were considered azoospermia and oligozoospermia, respectively.

### 2.2 Identification of HS cases and normospermatogenesis contrls

Azoospermic and oligozoospermic men with normal or slightly elevated follicle-stimulating hormone (FSH) levels (>11.95 mIU/mL) were advised for testicular fine needle aspiration (FNA) evaluation in order to be categorized as HS or normospermatogenesis (NS). The testicular FNA procedure, followed by reporting, was done at the Department of Pathology. In order to obtain the sample, a spermatic block containing 1% lignocaine was given to the HS patients. A 22-gauge needle attached to a 10 mL syringe on a plunger was used at two distinct locations to get the aspirates from the right and left testicles. A thread-like structure in the aspirate was used to prepare smears before staining. Every smear was confirmed for the presence of at least 2,000 cells. FNA from both testes shows a considerable decrease in the spermatogenic series of cells per Sertoli cell; however, maturation till spermatozoa is seen, was suggesting HS. The slide showing normal distribution of all stages of spermatogenic series of cell along with maturation till spermatozoa was suggestive of NS.

### 2.3 Identification of idiopathic HS cases

A comprehensive clinical history of the HS patients was taken to investigate male factor infertility. Patients were excluded if they had conditions such as the presence of hydrocele or varicocele, history of testicular trauma, testicular maldescent, and history of radiotherapy, chemotherapy, or exposure to any reproductive toxin and orchitis. A blood sample of 51 HS patients was collected in EDTA vials for DNA isolation using the QIAamp DNA Blood Mini Kit (QIAGEN, 51106) according to the manufacturer's instructions. The optical density of the isolated DNA was measured with a spectrophotometer (Nanodrop). Metaphase cell culture was conducted for 51 HS cases using a heparinized blood sample in karyotyping medium (Thermofisher Ref 12557-013). Fluorescence *in situ* Hybridization (XY FISH) analysis was performed on metaphase cells utilizing in-house X and Y centromere probes. The HS cases were considered idiopathic after excluding microdeletions of the Y chromosome found in 4 patients and aneuploidies of the sex chromosomes by FISH analysis, found on 5 patients (19). We found 42 idiopathic HS patients eligible for DNA methylation microarray study however two DNA samples failed the quality check mechanism to be considered for microarray, as a result of which the final inclusion consisted of 40 idiopathic HS and 10 NS DNA samples to be technically suitable for microarray analysis.

### 2.4 DNA methylation microarray

DNA was extracted from the residual testicular FNAC/biopsy samples using Qiagen's QIAamp DNA Mini Kit, following the manufacturer's protocol optimised for tissue samples. DNA from 40 patients with idiopathic HS and 10 NS was analyzed using the Illumina Infinium MethylationEPIC v2.0 BeadChip Kit (Cat. # 20087706). The target coverage was interrogates >935,000 CpG sites across the human genome, including high coverage of CpG islands, shores, shelves, promoters, enhancers, gene bodies, and ENCODE open chromatin regions. The workflow comprised of bisulfite conversion in which extracted genomic DNA was subjected to bisulfite treatment, which converts unmethylated cytosines to uracils, while methylated cytosines remain unchanged. The process of amplification and fragmentation is followed where bisulfite-converted DNA was then whole genome amplified and enzymatically fragmented. The process of hybridisation and extension was performed in which DNA samples were hybridised to array probes specific to CpG sites. A single-base extension was carried out using labelled nucleotides (dinitrophenyl or biotin-labelled ddNTPs). Arrays were stained with fluorescent markers and scanned using the Illumina iScan system. Signal intensities from methylated and unmethylated probes were used to calculate *β*-values representing methylation percentages. The scan platform used was Illumina iScan System using the *iScan Control Software (ICS)* to generate raw two-color IDAT files with green and red channels. We used GRCh38/hg38 reference genome build. In order to perform initial data scanning and analysis, Genome-studio 2.0 version was used. Raw intensities are corrected for background noise and dye by using their internal normalization methods in genome-studio software. *β*-values for each sample was calculated using the formula, average beta value=(Signal methylated)/(Signal methylated + Signal Unmethylated + 100). For quality control, a sample-level quality-control report was generated using the minfi::qcReport function, with samples grouped according to clinical condition. Detection *p*-values were calculated using detectionP(rgSet, type = “m + u”), and the mean detection *p*-value was assessed for each sample. Samples with a mean detection *p*-value >0.05 were flagged as failed. For probe-level filtering, probes were retained only when the detection *p*-value was <0.01 in all samples. Probes containing known SNPs were removed using dropLociWithSnps, and probes mapping to sex chromosomes (chrX/chrY), according to the EPICv2 hg38 annotation, were excluded. Differential methylation was assessed using a limma-based linear model on *β*-values, with a design matrix containing condition indicators (∼0 + Condition). Contrasts were constructed for group comparisons, followed by empirical Bayes moderation using eBayes. *P*-values were adjusted for multiple testing using the Benjamini–Hochberg false discovery rate (FDR). Differentially methylated probes (DMPs) were identified using limma-based empirical Bayes t-statistics, with significant DMPs defined as FDR-adjusted *p* < 0.05. Differentially methylated regions (DMRs) were identified using DMRcate (v2.0+). Significant CpGs were first annotated using cpg.annotate with an FDR < 0.05, followed by DMR identification using dmrcate with lambda=1000 and C = 2. Genomic ranges of the identified DMRs were subsequently extracted using extractRanges(genome="hg38”).

### 2.5 Data visualization and in-silico analysis

The heatmap illustrating TSS-associated differentially methylated spermatogenesis genes was generated in RStudio (R Foundation for Statistical Computing, Vienna, Austria) using the ggplot2 package. Gene-wise methylation values were normalized as row-wise Z-scores to facilitate comparison across samples, and the heatmap was constructed to visualize differential methylation patterns among idiopathic HS patients. To investigate the biological significance of differentially methylated genes, Gene Ontology (GO) enrichment and transcription factor protein–protein interaction analyses were performed using the Enrichr web-based platform. Lists of differentially methylated genes were submitted to Enrichr, and significantly enriched transcription factor interaction networks were identified using the available transcription factor gene-set libraries. The Enrichment Analysis Visualizer calculated term frequency–inverse document frequency (TF-IDF) values for the gene sets, followed by UMAP dimensionality reduction. Terms were positioned according to the first two UMAP dimensions, with proximity representing similarity in gene-set composition. Clusters were automatically identified using the Leiden algorithm applied to the TF-IDF values. A flowchart depicting analytical workflow of the study is shown in supplementary Figure 1.

### 2.6 Statistical data analysis

Clinical and laboratory data were analysed using GraphPad Prism (version 9.0, GraphPad Software, San Diego, CA, USA). Continuous variables are presented as mean ± standard error of the mean (SEM). Comparisons between the hypospermatogenesis (HS) and normospermatogenesis (NS) groups were performed using an unpaired Student's *t*-test. A *P* value of <0.05 was considered statistically significant. Statistical analysis for methylation burden was performed using a robust median absolute deviation (MAD)-based approach. The number TSS-associated DMRs identified in each patient was used to calculate the cohort median and MAD. Patients were subsequently classified into low-, intermediate-, and high-methylation burden groups based on predefined thresholds derived from the median and MAD values (low burden: < median; intermediate burden: median to median + 2.5 × MAD; high burden: > median + 2.5 × MAD). Descriptive statistics were used to summarize the distribution of hypermethylated and hypomethylated DMRs across the burden groups.

## 2 Results

### 3.1 Characteristics of the study cohorts

The reproductive hormonal profile, age, duration of infertility and testicular volume were evaluated across the study groups to identify statistically significant differences, particularly focusing on HS as cases and NS as control groups. No significant difference was found in age, duration of infertility and testicular volume of two study groups. Follicle stimulating hormone (FSH) levels were significantly elevated in the HS patients (11.20 ± 1.5 mIU/mL) compared to NS (5.39 ± 0.68 mIU/mL; *P* = 0.0191). This elevation supports the presence of impaired spermatogenic function in the HS group, consistent with the clinical diagnosis. Leutenising hormone (LH) levels also showed a trend toward elevation in HS subjects (6.9 ± 1.04 mIU/mL), compared to NS (3.7 ± 0.31 mIU/mL). These finding supports a compensatory increase in LH possibly due to Leydig cell dysfunction. Mean serum testosterone levels were significantly lower in the HS group (4.01 ± 0.3 ng/mL) relative to NS (5.4 ± 0.53 ng/mL; *P* = 0.0194). The mean serum values of FSH, LH, and testosterone for HS and NS remained within the expected clinical reference ranges.

### 3.2 Differential DNA methylation analysis

The DNA methylation microarray raw data was analysed for studying differential methylation pattern in terms of beta value in HS patients (Supplementary Figure 2). Figure 1 provides a descriptive overview of the genome wide distribution of DMRs in overall and individual HS patients. A majority of DMRs (80.57%) are located in unannotated regions (labelled as “Blank”), while 9.81% of DMRs fall within promoter regions, and 9.61% are associated with non-promoter regulatory elements (Figure 1A). Although the proportion of promoter-linked DMRs is lower, these are likely to be of higher regulatory relevance, directly impacting gene transcription. Majority of DMRs (60.85%) are located within CpG islands, on the other hand 30.96% of DMRs occur in shore regions, and a smaller proportion are found in shelf (7.08%) and open sea (1.08%) regions (Figure 1B). As per the University of California, Santa Cruz (UCSC) reference grouping, largest proportion of DMRs are annotated near transcription start sites (34.28%) within 200 bp (TSS200) and 29.81% within 1,500 bp (TSS1500) upstream of the TSS (Figure 1C). The DMR distribution indicates that methylation changes in HS are predominantly localised to CpG-rich promoter and upstream regulatory regions, with functional implications in gene expression control (Supplementary Figures 3a,b). We analysed the number and distribution of promoter-associated DMRs in testicular samples from individual idiopathic HS patients, mapped to UCSC reference regions including TSS, Exon 1, 5′UTR, and 3′UTR (Figure 1D). The majority of promoter associated DMRs localise to Exon 1 and TSS, with select patients such as HS2, HS5, HS8, HS10, and HS28 showing markedly higher DMR counts. Patients marked with an asterisk (*) indicate no filter retained DMRs, suggesting insufficient methylation variation to meet statistical thresholds in those individuals.

**Figure 1:**
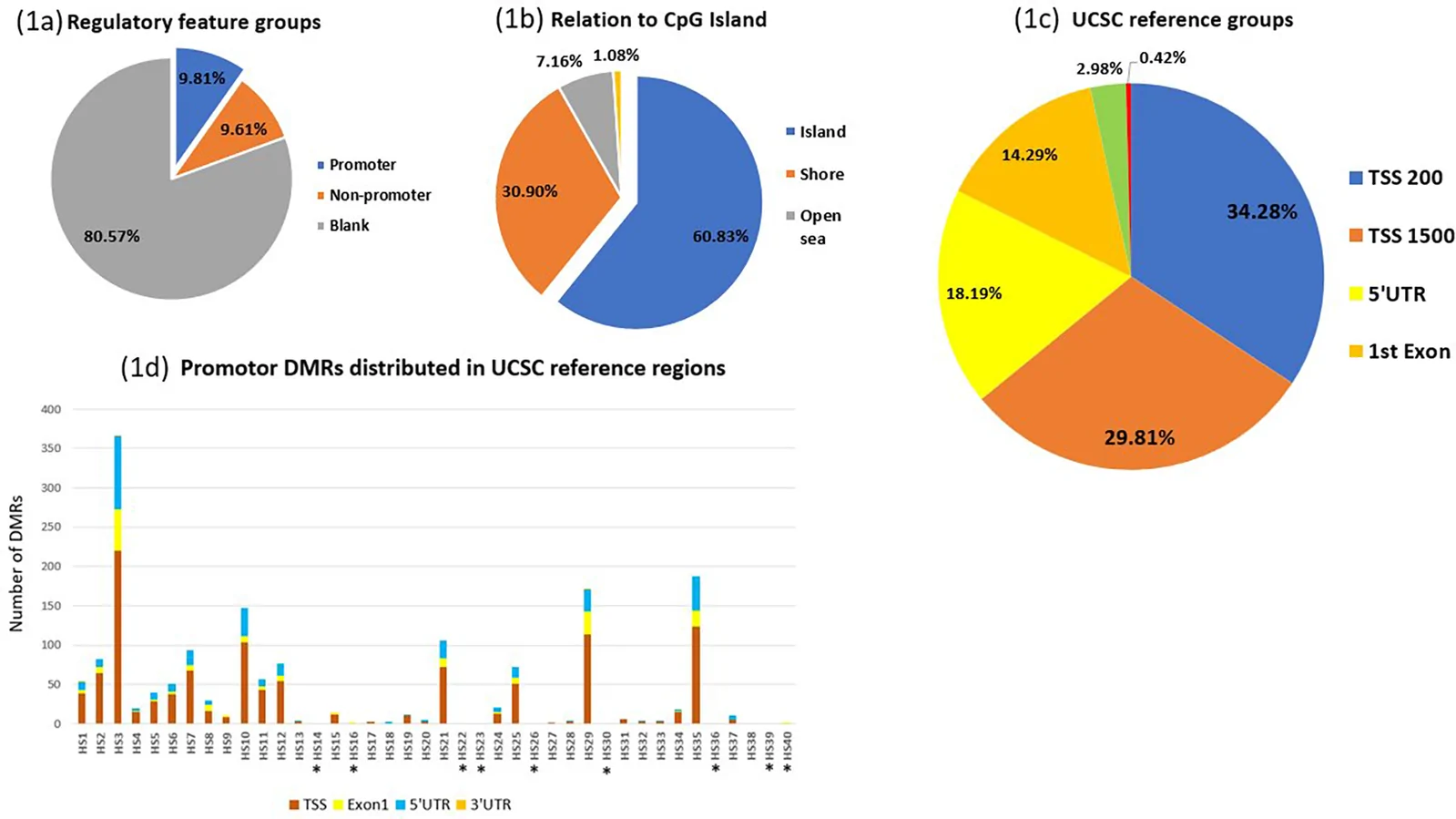
Genomic distribution of DMRs based on microarray data: **(a)** Regulatory feature groups; **(b)** Relation to CpG island; **(c)** University of California, Santa Cruz (UCSC) reference groups **(d)** Distribution of promoter-associated DMRs across UCSC reference regions.

### 3.3 Identification of spermatogenesis genes located in TSS-associated DMR

We filtered the spermatogenesis genes that are located in TSS-associated DMRs and assessed the average methylation levels of all those genes. To visualize inter-individual variation in methylation patterns, row-wise Z-score-transformed methylation values for spermatogenesis-associated genes were plotted across the 29 HS patients (Figure 2). The heatmap demonstrated heterogeneous methylation patterns across patients, with individual genes showing relative differences in methylation across the cohort.

**Figure 2:**
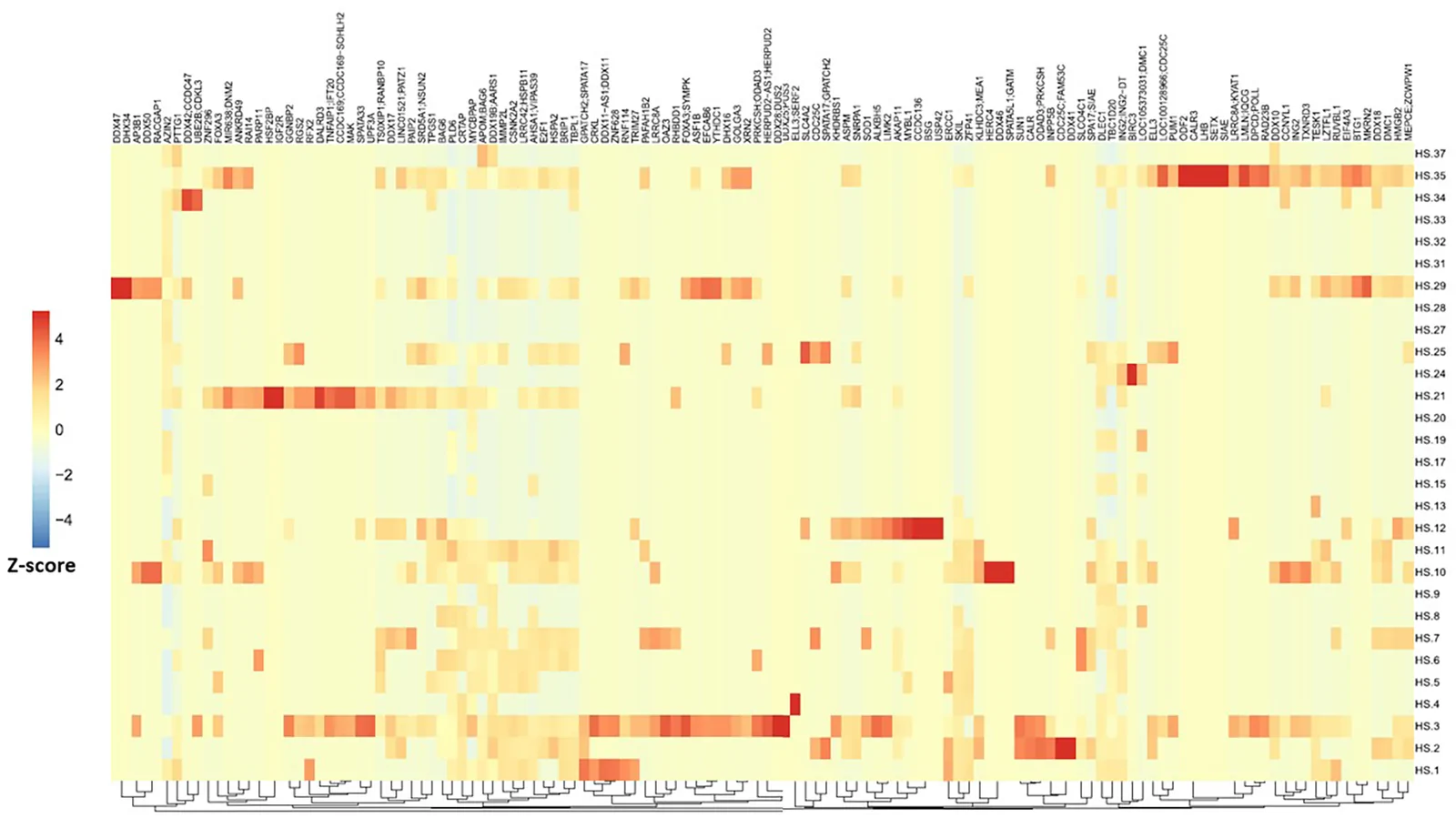
Relative methylation patterns of spermatogenesis-associated genes across the 29 HS patients using row-wise Z-score-transformed methylation values. (Colours indicate the row-wise Z-score normalised average expression levels, where blue indicates reduced expression, and red indicates elevated expression relative to the gene's mean).

Only genes with statistically significant differential methylation (padj ≤ 0.05) are included, ensuring biological relevance (Figure 2, Supplementary Figure 1). These data highlight the heterogeneity of TSS methylation patterns in HS, with hypermethylation of spermatogenesis genes being the more prevalent alteration. Gene ontology analysis was performed using the set of DMR associated genes identified in 29 HS patients (Supplementary Figures 4a–c).

### 3.4 Transcription factor protein-protein interaction analysis

To understand the impact of differential DNA methylation on, functional landscape of transcription factor, in-silico protein-protein interaction (PPI) enrichment analysis was performed. (Figure 3). The scatterplot displays all gene sets, with clusters indicating transcription factor groups that share enriched PPI terms. Larger black-outlined points represent significantly enriched transcription factor terms. Cluster 0 to 5 consisted of representative terms that are, TATA-box binding protein (*TBP*), Androgen receptor (*AR*), Specificity Protein 3 (*SP3*), Tumor Protein p53 (*TP53*), Cyclin T2 (*CCNT2*), and Transcriptional regulator (*SIN3A*).

**Figure 3:**
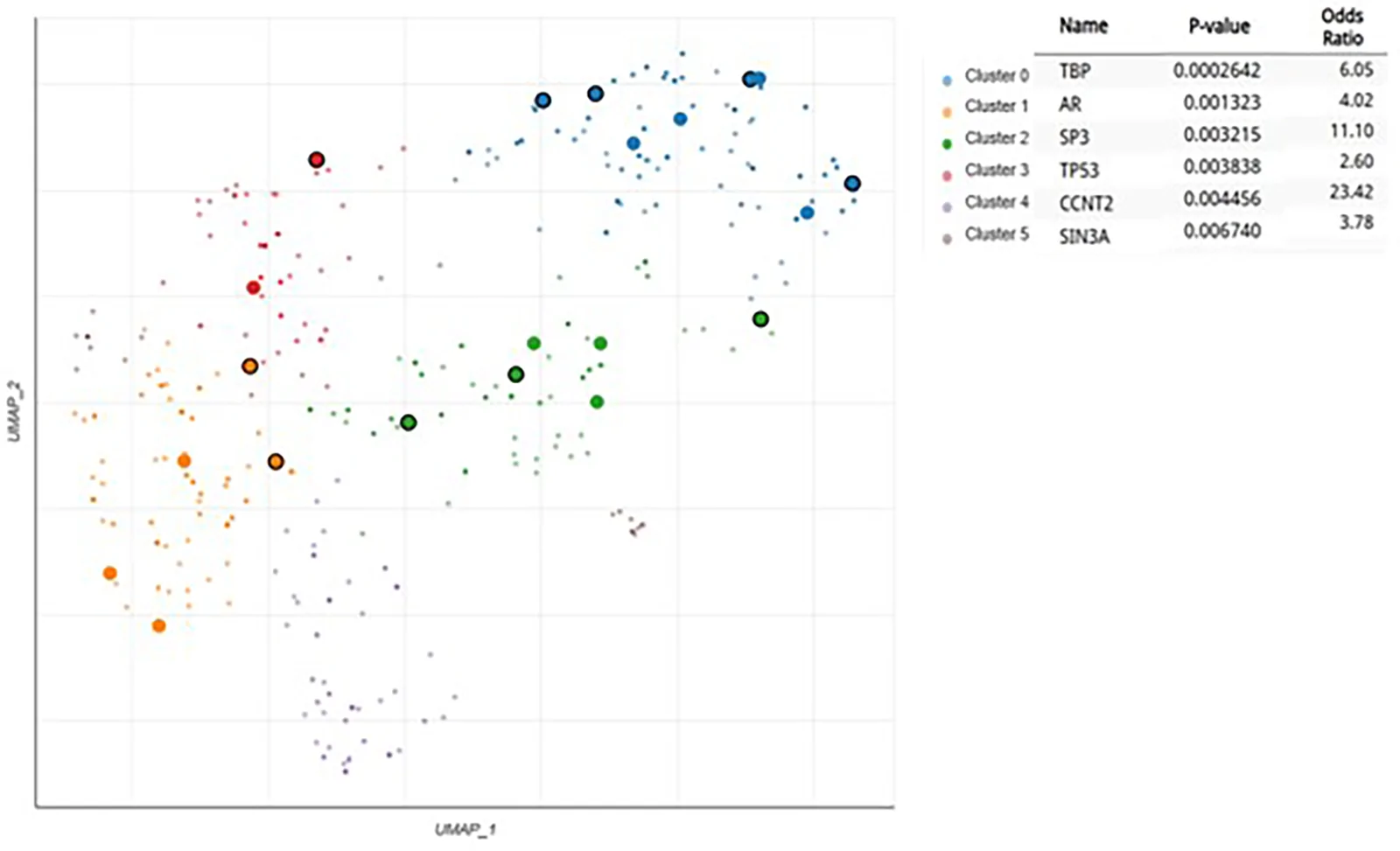
Scatterplot of all terms in the transcription factor PPIs gene set library.

### 3.5 Classification of patients as per DMR burden

To assess inter-sample variation in TSS-associated DMRs, a robust median absolute deviation (MAD)-based classification was applied. The median number of TSS-associated DMRs across the cohort found to be 37.5, with a MAD of 25. Based on these values, samples were stratified into low (<37.5), intermediate (37.5–100), and high (>100) methylation burden groups. Of the 29 samples analyzed, 20 (68.9%) were classified as low burden, indicating relatively fewer promoter-associated methylation alterations. Five samples (17.24%) fell within the intermediate burden category, representing moderate epigenetic perturbation, additionally four samples (13.7%) exceeded the median + 2.5 MAD threshold (>100 DMRs) and were categorized as high methylation burden, suggestive of substantial promoter-associated epigenetic dysregulation (Table 1). This stratification highlights marked inter-individual heterogeneity in TSS-associated methylation patterns within the cohort. To further characterize the directionality of methylation changes in TSS-associated DMRs, the total burden of hypermethylated and hypomethylated DMRs was assessed across both high- and intermediate-burden samples. A total of 851 TSS-associated DMRs were identified, of which 721 (84.7%) were hypermethylated and 130 (15.3%) were hypomethylated.

**Table 1:** List of idiopathic HS patients with significant burden of aberrantly methylenated spermatogenesis genes associated with DMRs.

Patient Id	Classification DMR burden	Hypo genes	Hyper Genes	Age (years)	FSH (mIU/mol)	LH (mIU/mol)	T. (nmol/L)	Biological process enrichment	Functional enrichment
HS3	High burden	28	191	30	6.7	3.45	4.43	GO:0007283-Spermatogenesis	GO:0008186-ATP-dependent activity on RNA
HS10	High burden	20	83	35	7.76	8.27	5.48	GO:0030154-Cellular differenctiation GO:0048869-Cellular developmental process	GO:0008186-ATP-dependent activity on RNA
HS29	High burden	12	102	31	5.7	4.12	3.27	GO:0007281-Germ cell development	GO: 0032553-Ribonucleotide binding
HS35	High burden	16	108	33	5.88	4.42	2.49	GO:0000278-Mitotic cell cycle, GO:0051726-Regulation of cell cycle	GO:0003677-DNA binding GO:0061629-RNA polymerase-2 specific DNA binding transcription factor binding
HS1	Intermediate	8	30	28	4.38	5.2	3.99	GO:0048323-Male gamete generation	Information not available
HS2	Intermediate	9	55	32	2.87	3.56	4.82	GO:0003006-Developmental process involved in reproduction	GO:0008186-ATP dependent activity on acting RNA
HS7	Intermediate	13	54	34	4.73	5.35	3.64	GO:0022414-Reproductive process	GO:0005515-Protein binding
HS21	Intermediate	13	58	36	7.59	4.37	5	GO:0048323-Male gamete generation	GO:0003729-mRNA binding GO:0005515-Protein binding
HS25	Intermediate	11	40	33	6.3	2.57	2.71	GO:0007283-Spermatogenesis	Information not available

Biological reference range: Follicle Stimulating Hormone (FSH): 0.95–11.95 mIU/mL; Luteinizing Hormone (LH): 0.57–12.07 mIU/mL; Serum Testosterone (T): 1.42–9.23 ng/mL; hypo = hypomethylated genes; hyper = hypermethylated genes; GO = Gene ontology.

## 4 Discussion

Spermatogenesis involves extensive epigenetic reprogramming, including DNA methylation changes that regulates gene expression. Primordial germ cells undergo genome-wide erasure of DNA methylation marks early in development, followed by establishment of new methylation patterns during male germ cell differentiation. Previous landmark studies have established the significance of epigenetic dysregulation in male infertility and laid the foundation for investigating DNA methylation signatures in azoospermia, where in the absence of sperm in the ejaculate has driven studies examining differential DNA methylation in testicular tissue or blood samples (20–22). Our study investigates differential DNA methylation using DNA derived from testicular tissue samples of patients with idiopathic HS. These patients have a relatively high likelihood of successful sperm retrieval and the potential to father their biological children through assisted reproductive techniques. Therefore, characterizing the epigenetic alterations and their potential heritability in this group is of particular importance.

In the current study a genome-wide DNA methylation profiling in testicular tissue from 40 HS patients identified 1,666 DMRs localised predominantly at promotor (transcriptional regulatory) regions. A significant proportion (70%) of these DMRs exhibited hypermethylation, suggesting widespread transcriptional repression of spermatogenesis-associated genes. Our data corelates well with the findings of Ferfouri F. et al. who demonstrated widespread aberrant methylation patterns associated with impaired spermatogenesis, highlighting the contribution of epigenetic instability to testicular dysfunction (15). At the same time findings of the current study corelates with Sarkar S. et al. who identified differential methylation signatures in peripheral blood (23). Together, these studies provided important evidence that DNA methylation is closely linked to male reproductive pathology at testicular tissue and blood level. The present study revealed distinct hypo- and hypermethylation signatures in genes associated with spermatogenesis, suggesting a widespread epigenetic imbalance in NOA. Hypomethylated DMR-associated genes were enriched for several key regulators of germ cell development, including *TAF4B, RFX2, CREM, YTHDC2, TDRKH*, and *PLD6,* all of which are known to play critical roles in meiotic progression, piRNA biogenesis, and spermiogenesis. Aberrant hypomethylation in these loci may reflect dysregulated activation of germ cell-specific transcriptional programs, potentially contributing to impaired testicular function. In contrast, hypermethylated DMRs were associated with genes involved in meiotic chromosome dynamics, DNA repair, and epigenetic regulation, such as *STAG3, SYCE2, TCFL5, DNMT1, SETDB1*, and *UHRF1*. Hypermethylation of these genes may result in transcriptional repression, thereby disrupting meiotic integrity, chromatin organization, and genome stability. Notably, several recurrent genes identified in this study, including *KDM3A, CFAP69*, and *SPATA* family members, have established roles in male fertility, further supporting the biological relevance of our findings.

Previous studies have identified aberrant methylation in several spermatogenesis-associated genes, including *MTHFR, DDR1, SPATA16, CNGA1, MAEL, BOLL, DDX4*, and *SOX30* in NOA patients (11, 24) Comparative analysis of our idiopathic HS cohort revealed partial overlap with these findings. Notably, we identified differential methylation in *CNGA1,* a gene previously implicated in gamete formation and spermatogenic defects. Additionally, while direct methylation changes in *MTHFR* were absent, related folate pathway genes such as *MTHFD1* and *MTHFD1L* were observed, suggesting possible disruption of one-carbon metabolism. Similarly, although canonical piRNA-pathway genes such as *MAEL* and *DDX4* were not detected, several RNA helicases and germline regulatory genes including *YTHDC2* and *DDX17* were differentially methylated, indicating alternative epigenetic mechanisms contributing to spermatogenic dysfunction in HS. No direct overlap with the classical imprinted genes (*H19, IGF2,* and *MEST*) was observed in our HS cohort. However, several genes involved in epigenetic maintenance *(DNMT1, SETDB1, EZH1, CHD4),* DNA repair *(RAD21, RAD51, TP53BP1),* and germline development (*YTHDC2, MEIG1, SPATA* genes) were differentially methylated, suggesting that while canonical imprinting defects may not be central in your cohort, broader epigenetic regulatory mechanisms remain significantly disrupted.

The studies conducted so far have explored DNA methylation changes in male infertility, most have focused on individual genes, imprinted regions, or global methylation patterns. However, the specific epigenetic regulation of gene networks particularly those involved in transcription factor protein– protein interactions, remains largely unexplored. The genome-wide DNA methylation study by Ferfouri F. provided early evidence of widespread epigenetic dysregulation in testicular samples from NOA patients, implicating aberrant methylation as a contributor to impaired spermatogenesis. Importantly, the authors emphasized the need for integrating DNA methylation findings with mRNA expression profiling and immunohistochemical validation to better establish the functional consequences of these epigenetic alterations (15). Building upon this framework, the present study extends that recommendation by systematically correlating differential methylation with gene expression patterns in testicular tissue by further integrating in silico network-based analyses, including and regulatory clustering. Using in-silico transcription factor protein-protein interaction analysis we identified six clusters viz., *TBP, AR, SP3, TP53, CCNT2* and *SIN3A*, indicating their potential role in the epigenetic mechanisms of the condition. The *TBP* plays a crucial role in gene transcription by initiating the assembly of the transcription machinery at gene promoters. In the context of male fertility, TBP's function extends beyond its general role in transcription, research indicates that *TBP* expression is significantly upregulated during spermatogenesis. In both rat and mouse models, *TBP* mRNA levels were found to increase more than 40-fold in the testis and spermatogenic cells compared to somatic tissues. This upregulation is stage-dependent, occurring specifically in meiotic and post-meiotic cells, suggesting that *TBP* is essential for the transcriptional regulation during these critical stages of sperm development (25). Balagannavar G. and colleagues performed transcriptomic profiling in NOA and identified dysregulated genes involved in spermatogenesis, testicular development, and transcriptional control, which aligns with our PPI-based identification of key transcription-associated hubs such as *TBP* (26). Hypermethylation of the *SP3* gene, which encodes the Sp3 transcription factor, can significantly impact male fertility by disrupting critical transcriptional networks involved in spermatogenesis (27). On the other hand, hypermethylation of the *TP53* gene, can significantly impact male fertility by disrupting transcription factor interactions and triggering apoptotic pathways in germ cells (28). Hypermethylation of the *CCNT2* gene, which encodes Cyclin T2, can significantly impact male fertility by disrupting transcription factor interactions and affecting spermatogenesis. Cyclin T2 is a regulatory subunit of the positive transcription elongation factor b (P-TEFb) complex, which includes *CDK9* (29). This complex is essential for the phosphorylation of the RNA polymerase II C-terminal domain, facilitating transcription elongation. Hypermethylation of the *SIN3A* gene, which encodes the transcriptional corepressor Sin3A, can significantly impact male fertility by disrupting transcription factor interactions and affecting spermatogenesis. Sin3A is expressed in Sertoli cells, where it plays a role in establishing a niche for undifferentiated spermatogonia and supporting spermatid elongation. Studies have shown that Sin3A is required in Sertoli cells to establish a niche for undifferentiated spermatogonia and support spermatid elongation (30).

We performed a median absolute deviation-based classification of our idiopathic HS patients based on the number of hypo and hyper methylated genes in TSS-associated DMRs. We compared the DMR-associated genes identified in our high-burden and intermediate-burden patient groups with previously reported methylation signatures in NOA. Interestingly, the high-burden group demonstrated a considerable overlap with hypermethylated genes reported by F. Ferfouri et al. and Y. S. Cheng et al., suggesting a shared epigenetic disruption pattern associated with impaired spermatogenesis (11, 15). Among the overlapping hypermethylated genes were *JAK1, BCL11B, SMAD6, REST, HIF1A, SOCS1, TP53BP1, MLH3, FANCM, RAD21, CDC6, RAD51, UHRF1, DNMT1, CDKN2A, E2F2, E2F8, FOXM1, BUB1, CENPA, CENPE, KIF11, KIF14, UBE2C, UBE2 T, CDK1*, and *HJURP,* many of which are implicated in cell-cycle regulation, DNA damage repair, chromatin remodeling, and meiotic progression. This overlap reinforces the hypothesis that aberrant hypermethylation of spermatogenesis-related and genome-maintenance genes may contribute to germ cell arrest and testicular dysfunction in NOA. Furthermore, the enrichment of genes involved in checkpoint regulation and DNA repair pathways in our high-burden group suggests a potentially more severe epigenetic dysregulation compared to intermediate-burden patients, which may underlie the greater extent of spermatogenic impairment observed in these individuals. These observations collectively suggest that defective spermatogenesis in NOA may arise from a dual epigenetic mechanism involving both aberrant activation and silencing of critical reproductive pathways.

## 5 Conclusions

Clinical utility of DNA methylation profiling in idiopathic male infertility is still emerging. The present findings should be interpreted as preliminary and exploratory rather than as an immediately applicable diagnostic or prognostic tool. The methylation alterations cannot currently be used independently for diagnosis, prognosis, or clinical management, they may provide a foundation for the identification and validation of DNA methylation episignatures in larger and independently validated cohorts. In our study the DMRs identified across TSS in idiopathic HS patients, highlight the heterogeneity of TSS methylation patterns in HS, with hypermethylation of spermatogenesis genes being the more prevalent alteration. However, the presence of hypomethylation in a substantial number of patients suggests complex, bidirectional epigenetic dysregulation contributing to spermatogenic failure.

## 6 Limitation

The relatively small cohort size, exploratory nature of the analysis, and lack of an independent validation cohort limit the immediate clinical applicability and generalizability of the identified differentially methylated regions. Furthermore, the functional consequences of these methylation alterations require experimental validation. Therefore, our findings should be regarded primarily as exploratory. As a technical limitation we highlight that cell-type deconvolution or adjustment for cellular composition was not performed in the present study. Therefore, our findings represent tissue-level DNA methylation associations and should not be interpreted as cell-type-specific epigenetic alterations. A potential batch effects associated with this analysis could not be adequately assessed. Therefore, the patient-specific DMR findings should be interpreted as exploratory.

## Data Availability

The IDAT files for all patients are available and may be shared upon reasonable request to the corresponding author, subject to a valid scientific justification.
