# Supplementary material for: Differential DNA methylation profiling of spermatogenesis-associated genes in patients with idiopathic hypospermatogenesis: a hospital-based case-control study
Source: Front Reprod Health. 2026 Oct 28;8:1932019. doi: 10.3389/frph.2026.1932019 (PMC13752042; doi:10.3389/frph.2026.1932019)
Supplement: Supplementary file 1 [file Datasheet1.pdf]

Supplementary figure 1. Analytical workflow of the study

STUDY COHORT
40 HS patients + 10 NS controls

↓

TESTICULAR FNA-DERIVED DNA
Residual FNA material after routine pathological slide preparation

↓

DNA METHYLATION PROFILING
Illumina Infinium MethylationEPIC v2.0 BeadChip (>935,000 CpG sites) → iScan scanning → IDAT files

↓

DATA PROCESSING & QUALITY CONTROL
GenomeStudio 2.0: background correction, dye-bias correction/internal normalization, probe/sample QC and batch-effect assessment

↓

PRIMARY DIFFERENTIAL METHYLATION ANALYSIS
40 HS vs 10 NS → differential methylation analysis → statistically significant DMRs (FDR/padj ≤ 0.05)

↓

PATIENT-SPECIFIC DMR ANALYSIS
Each HS patient compared with the same 10 NS reference samples; patient-specific DMRs retained using padj ≤ 0.05

↓

29 HS patients<br>≥1 statistically significant DMR retained	11 HS patients<br>No statistically significant DMR retained<br>Not excluded from primary analysis; not used for DMR-dependent downstream analyses

↓

TSS-ASSOCIATED DMR ANNOTATION & SPERMATOGENESIS-GENE SELECTION (29 HS)
DMRs mapped to GRCh38/hg38 UCSC regions (TSS, Exon 1, 5′UTR, 3′UTR) → spermatogenesis-associated genes identified → significant genes selected (padj ≤ 0.05)

↓

FIGURE 2: PATIENT-LEVEL METHYLATION HEATMAP<br>Selected spermatogenesis-associated genes → underlying patient-level methylation values for 29 HS patients → row-wise Z-score transformation → heatmap showing relative methylation patterns/inter-individual heterogeneity<br><br>Interpretation: descriptive visualization; Z-scores do not independently define HS-vs-NS hyper/hypomethylation.	PATIENT-SPECIFIC GO ANALYSIS<br>For each HS patient, genes associated with that patient's statistically significant DMRs were analyzed separately using g:Profiler → GO enrichment<br><br>Purpose: exploratory in-silico functional annotation/hypothesis generation.	TF–PPI / ENRICHR ANALYSIS<br>Genes represented in Figure 2 heatmap → Enrichr Transcription_Factor_PPIs library → enrichment analysis → TF-IDF → UMAP → Leiden clustering<br><br>Figure 3: computational clustering of enriched gene-set terms; not experimental TF–DNA/PPI evidence.

Supplementary figure 2: Assessment of differential methylation

The density plots showing the distribution of DNA methylation β-values in testicular tissue samples from idiopathic hypospermatogenesis (HS) patients and normal spermatogenesis (NS) controls, both before (raw) and after (normalized) preprocessing. In the raw data panel (Fig. 2a), the β-value distributions vary across samples, particularly between HS and NS groups, indicating batch effects and technical variability. Although the bimodal distribution typical of methylation microarray data is evident, inconsistent density peaks suggest the need for normalization to correct for technical noise and ensure comparability across datasets. After normalization (Fig. 2b), the β-value distributions become uniform across both HS and NS groups. The alignment of curves and the preservation of bimodal peaks (at $\beta \approx 0.1$ for unmethylated and $\beta \approx 0.9$ for fully methylated sites) confirm effective correction of inter-sample variability. This enhances the accuracy and reliability of downstream differential methylation analysis.

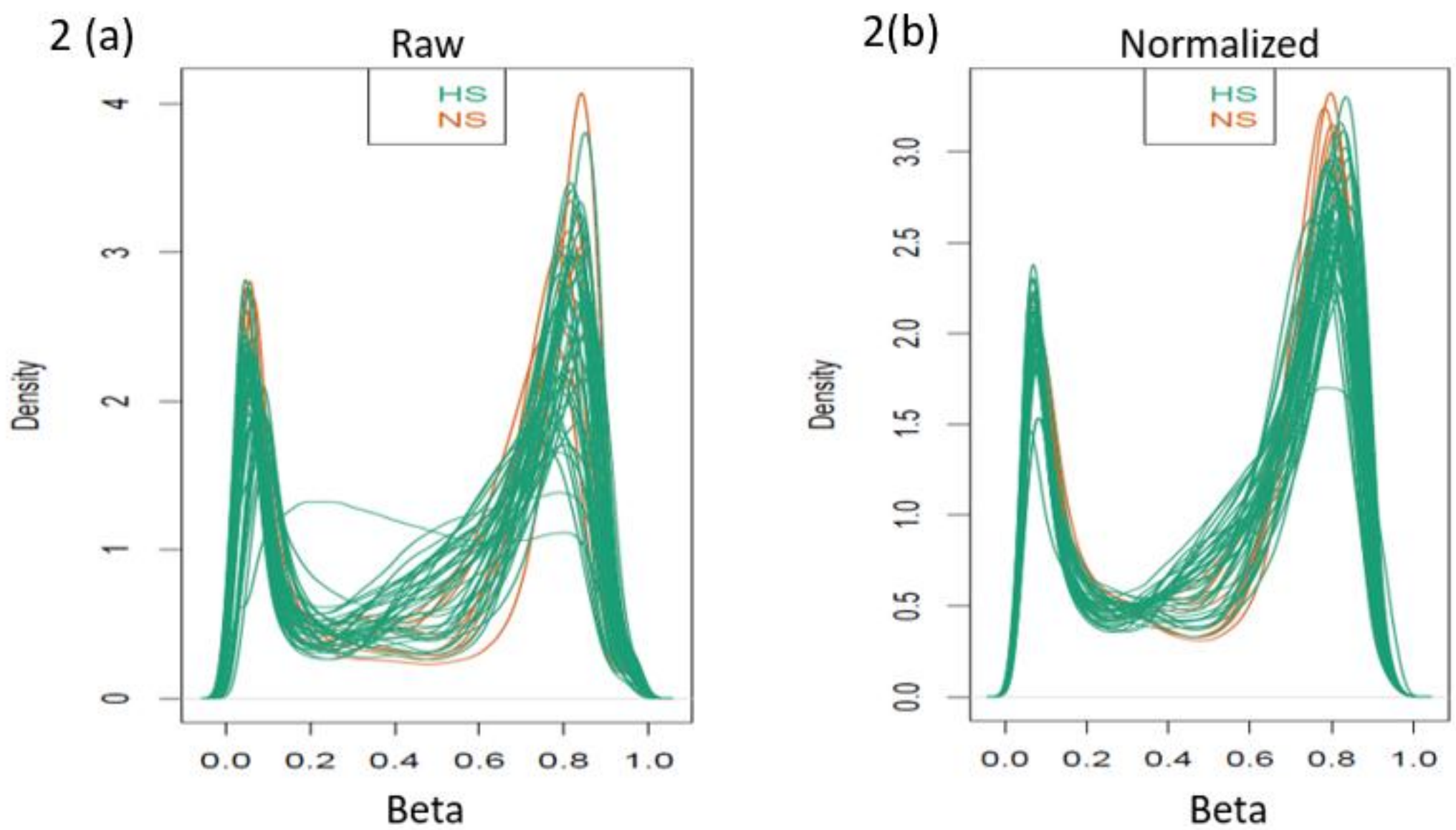

Supplementary figure 3: Distribution of DMRs across three promoter-associated UCSC reference regions

The bar graph is depicting TSS, 5′UTR, and Exon 1 associated DMRs in 40 idiopathic HS patients. The DMRs are categorised as either hypomethylated (blue) or hypermethylated (orange). The majority of promoter DMRs localise to TSS regions, with a striking predominance of hypermethylation (~70%), suggesting transcriptional repression of genes involved in spermatogenesis (pie chart). Both the 5′UTR and Exon 1 regions also show similar patterns, although the number of DMRs is lower.

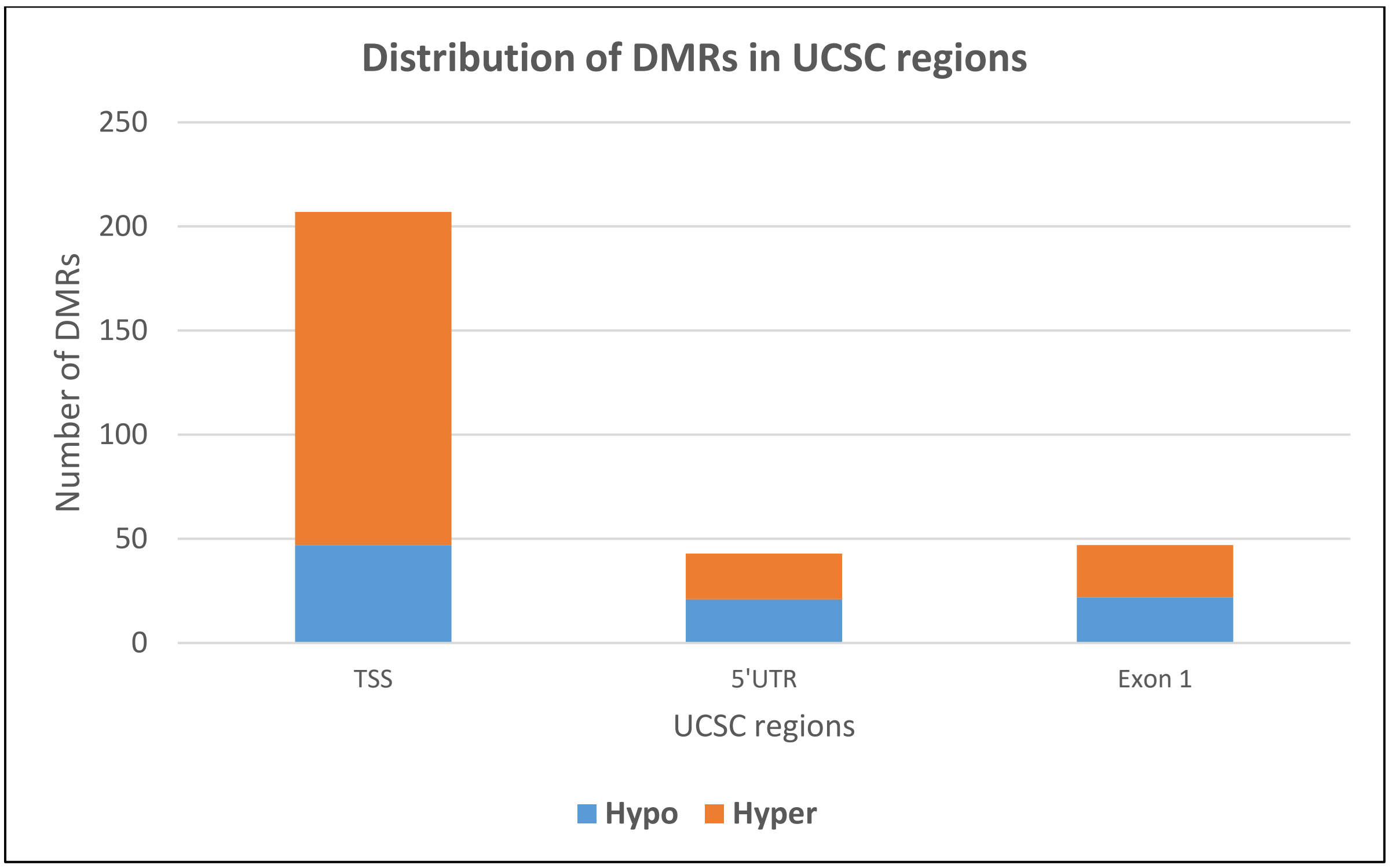

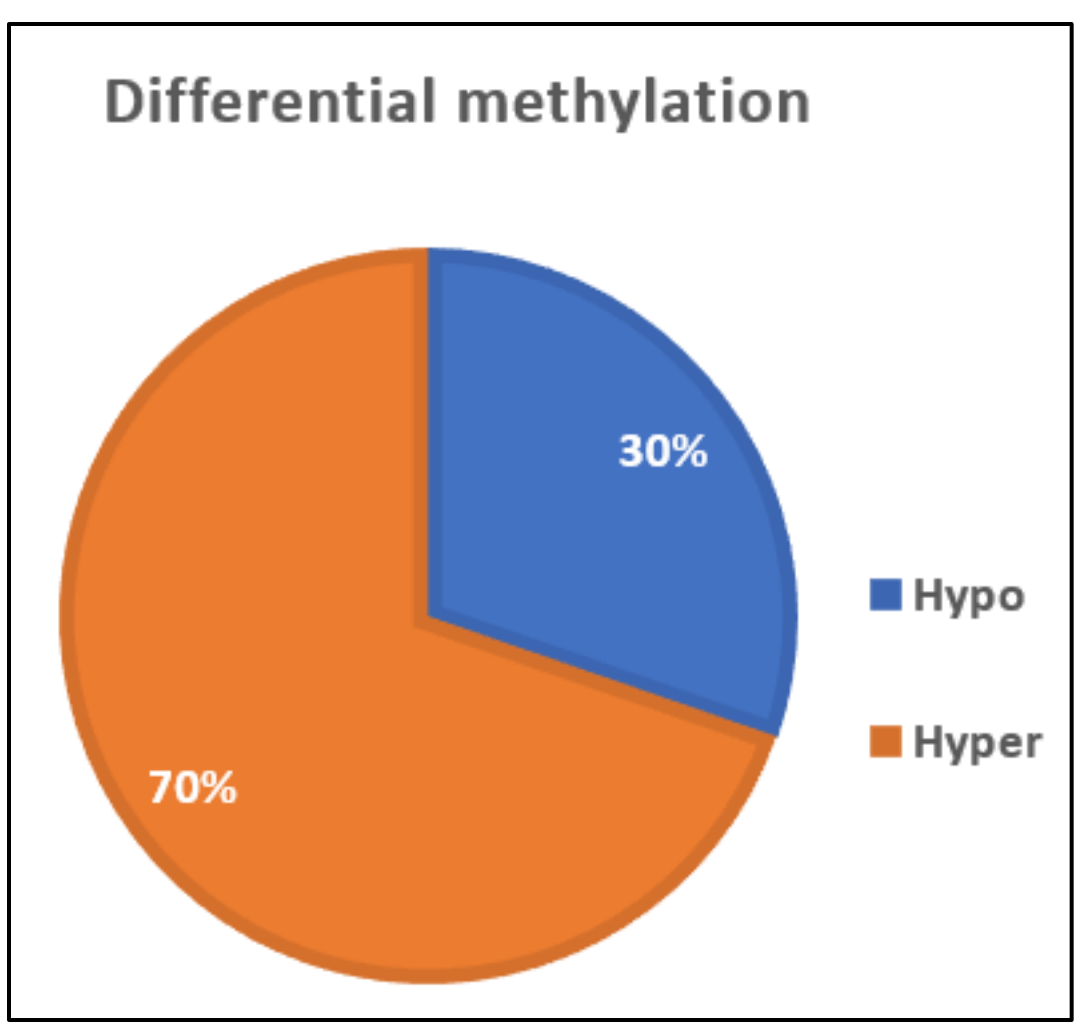

Supplementary figure 4: Gene ontology analysis

The figures 3a,3b and 3c are representing GO-based enrichment analysis of genes associated with promoter DMRs in HS, classified by molecular function, biological process, and cellular component. Molecular Function (Fig.4a): The most significantly enriched functions include Protein binding (GO:0005515), RNA binding (GO:0003723), ATP binding (GO:0005524), and DNA binding (GO:0003677). These functions indicate that methylation changes in promoter regions are affecting genes involved in transcriptional regulation, ATPdependent enzymatic activity, and chromatin dynamics all of which are essential for germ cell maintenance and transcriptional fidelity during spermatogenesis. Biological Process (Fig. 4b): Notably enriched biological processes include Spermatogenesis (GO:0007283), Cell differentiation (GO:0030154), Chromatin organisation (GO:0006325), and DNA repair and DNA damage response. The prominence of spermatogenesis and spermatid development in the top enriched terms directly supports the hypothesis that epigenetic disruption in regulatory elements contributes to the spermatogenic failure characteristic of HS. 111 Cellular Component (Fig. 4c): Genes were predominantly localised to Nucleus (GO:0005634), Cytoplasm (GO:0005737), Chromosome, centrosome, and nucleolus. These localisations align with functional requirements for germ cell maturation, cell cycle progression, and chromatin remodelling. The enrichment in nuclear compartments further supports the involvement of epigenetic regulatory machinery in HS pathology.

4 (a)

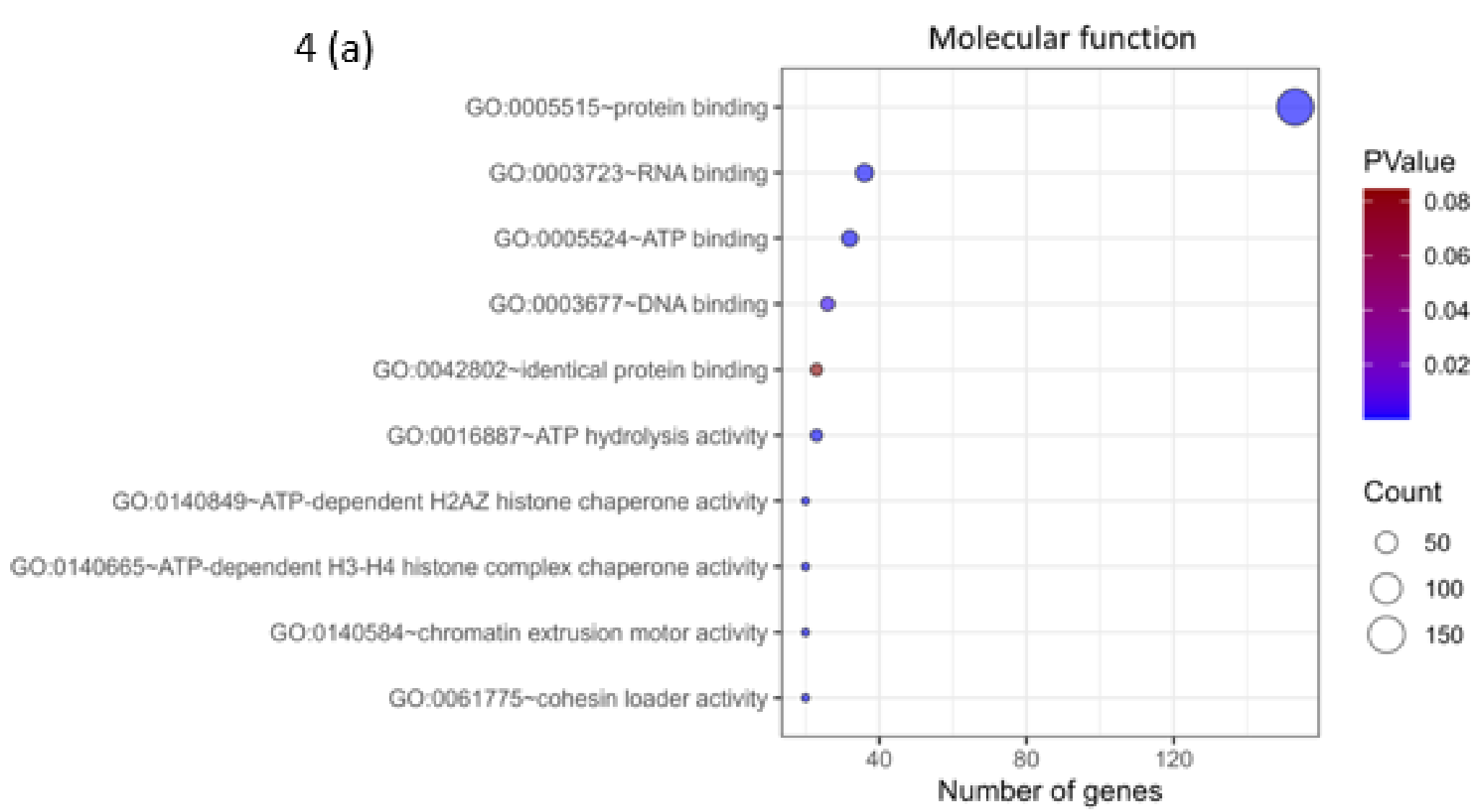

4 (b)

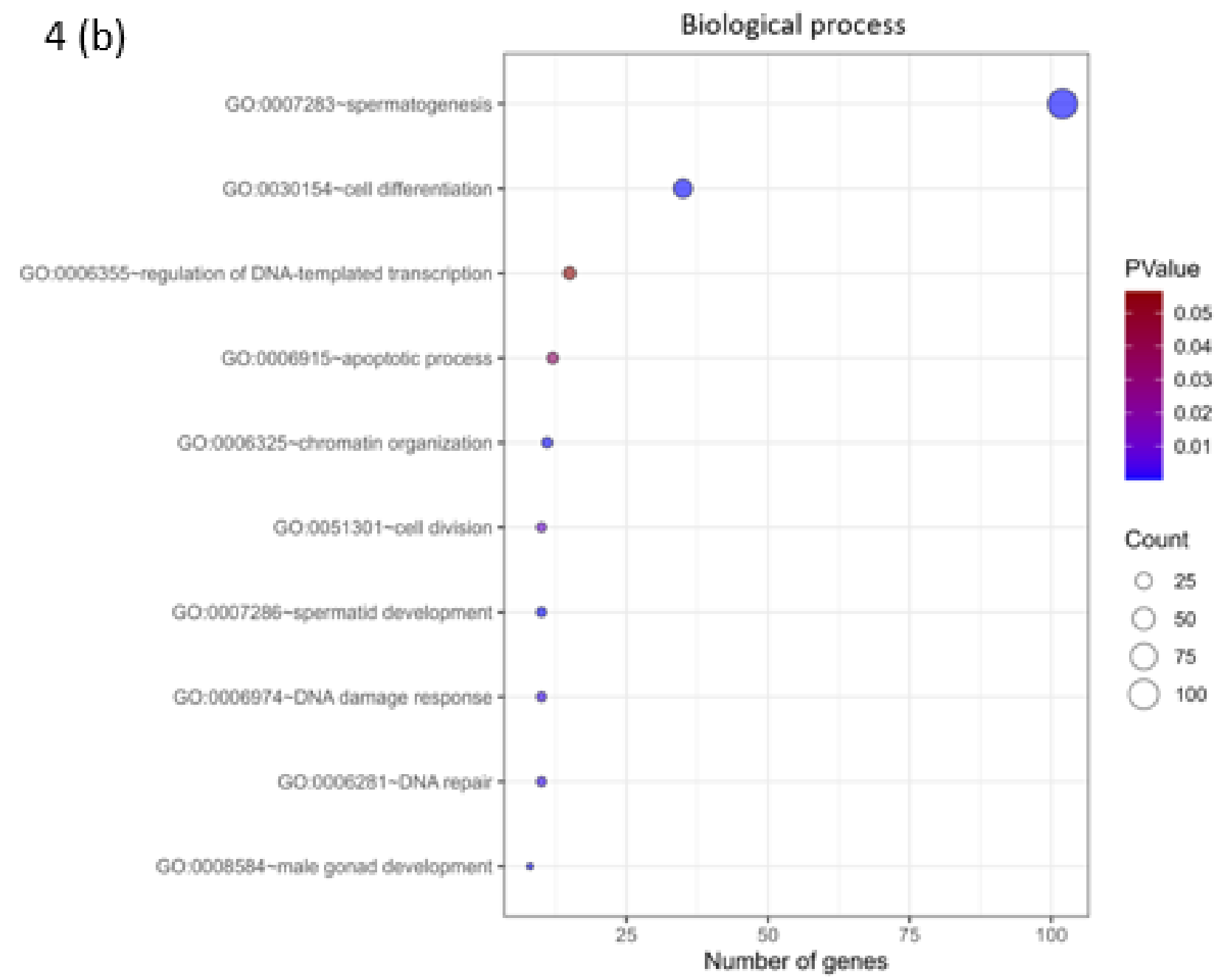
Biological process
GO:0007283~spermatogenesis
GO:0030154~cell differentiation
GO:0006355~regulation of DNA-templated transcription
GO:0006915~apoptotic process
GO:0006325~chromatin organization
GO:0051301~cell division
GO:0007286~spermatid development
GO:0006974~DNA damage response
GO:0006281~DNA repair
GO:0008584~male gonad development
25
50
75
100
Number of genes
PValue
0.05
0.04
0.03
0.02
0.01
Count
25
50
75
100

4 (c)

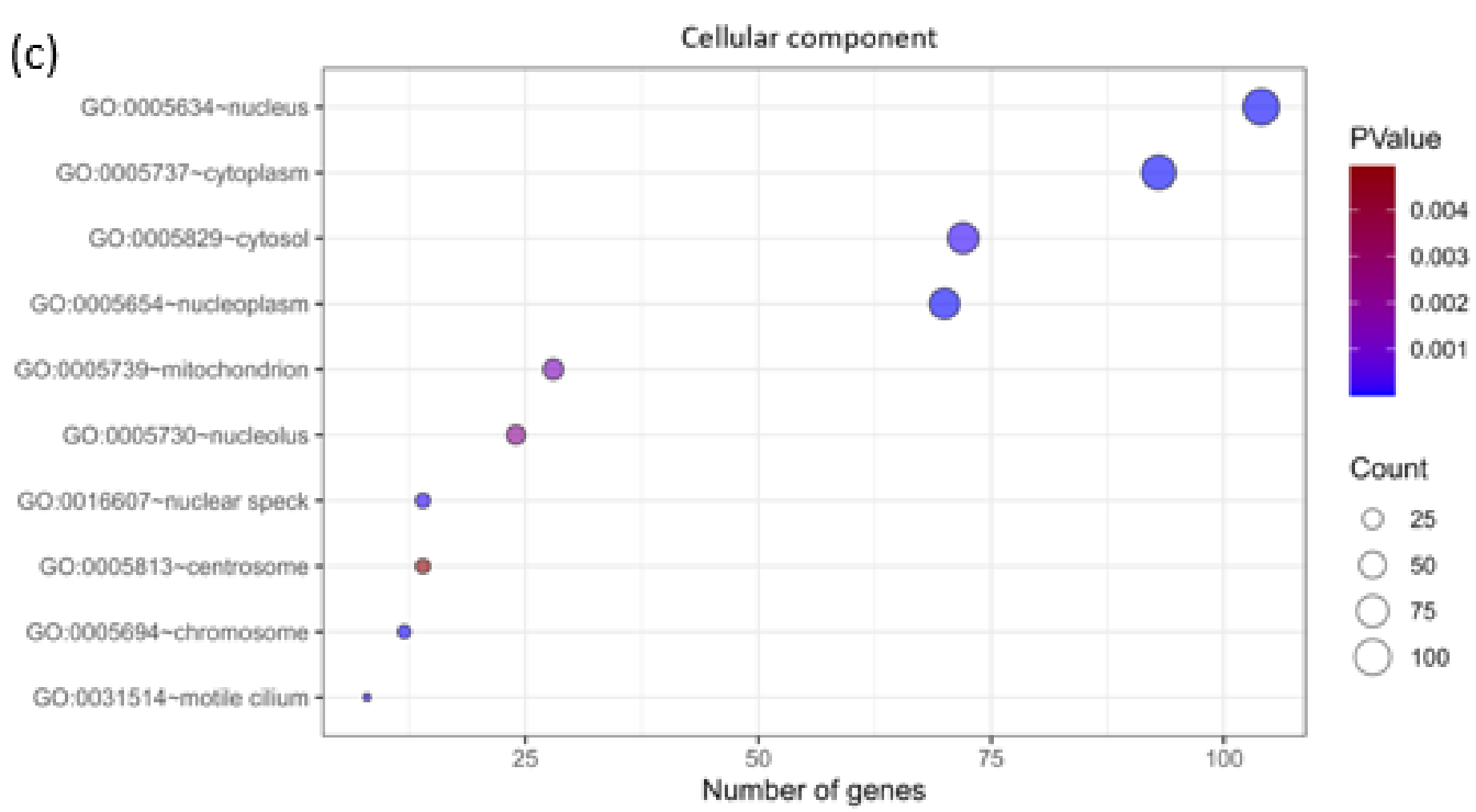
Cellular component
GO:0005634~nucleus
GO:0005737~cytoplasm
GO:0005829~cytosol
GO:0005654~nucleoplasm
GO:0005739~mitochondrion
GO:0005730~nucleolus
GO:0016607~nuclear speck
GO:0005813~centrosome
GO:0005694~chromosome
GO:0031514~motile cilium
25
50
75
100
Number of genes
PValue
0.004
0.003
0.002
0.001
Count
25
50
75
100
